# Extracellular matrix remodelling in response to venous hypertension: proteomics of human varicose veins

**DOI:** 10.1093/cvr/cvw075

**Published:** 2016-04-11

**Authors:** Javier Barallobre-Barreiro, Rahmi Oklu, Marc Lynch, Marika Fava, Ferheen Baig, Xiaoke Yin, Temo Barwari, David N. Potier, Hassan Albadawi, Marjan Jahangiri, Karen E. Porter, Michael T. Watkins, Sanjay Misra, Julianne Stoughton, Manuel Mayr

**Affiliations:** 1 King's British Heart Foundation Centre, King's College London, 125 Coldharbour Lane, London SE5 9NU, UK; 2 Division of Vascular and Interventional Radiology, Mayo Clinic, Scottsdale, AZ, USA; 3 St George's Hospital, NHS Trust, London, UK; 4 Division of Vascular Surgery, Massachusetts General Hospital, Harvard Medical School, Boston, MA, USA; 5 Institute of Cardiovascular and Metabolic Medicine, University of Leeds, Leeds, UK; 6 Division of Vascular and Interventional Radiology, Mayo Clinic, Rochester, MN, USA

**Keywords:** Venous, Extracellular matrix, Smooth Muscle, Protease

## Abstract

**Aims:**

Extracellular matrix remodelling has been implicated in a number of vascular conditions, including venous hypertension and varicose veins. However, to date, no systematic analysis of matrix remodelling in human veins has been performed.

**Methods and results:**

To understand the consequences of venous hypertension, normal and varicose veins were evaluated using proteomics approaches targeting the extracellular matrix. Varicose saphenous veins removed during phlebectomy and normal saphenous veins obtained during coronary artery bypass surgery were collected for proteomics analysis. Extracellular matrix proteins were enriched from venous tissues. The proteomics analysis revealed the presence of >150 extracellular matrix proteins, of which 48 had not been previously detected in venous tissue. Extracellular matrix remodelling in varicose veins was characterized by a loss of aggrecan and several small leucine-rich proteoglycans and a compensatory increase in collagen I and laminins. Gene expression analysis of the same tissues suggested that the remodelling process associated with venous hypertension predominantly occurs at the protein rather than the transcript level. The loss of aggrecan in varicose veins was paralleled by a reduced expression of aggrecanases. Chymase and tryptase β1 were among the up-regulated proteases. The effect of these serine proteases on the venous extracellular matrix was further explored by incubating normal saphenous veins with recombinant enzymes. Proteomics analysis revealed extensive extracellular matrix degradation after digestion with tryptase β1. In comparison, chymase was less potent and degraded predominantly basement membrane-associated proteins.

**Conclusion:**

The present proteomics study provides unprecedented insights into the expression and degradation of structural and regulatory components of the vascular extracellular matrix in varicosis.

## 1. Introduction

Venous hypertension is among the most prevalent medical problems today causing significant morbidity in a variety of clinical conditions including portal venous hypertension, haemorrhoids, pelvic congestion syndrome, nutcracker syndrome, varicose veins, and venous ulcers.^1^ While consequences of venous hypertension are apparent, its pathophysiology and chronic effects on the vessel wall are poorly understood, limiting preventative and therapeutic options. Among the spectrum of conditions associated with venous hypertension, varicose veins in the lower extremities are the most studied because of their high prevalence and easy access to tissue for analysis. Approximately 23% of adults in the USA alone have varicose veins with significant impact on the quality of life of the patients.^2^ There are numerous risk factors to the development of varicose veins including obesity, trauma, and pregnancy; however, genetic predisposition is probably the most important factor.^3^

Chronic venous reflux can lead to structural changes that ultimately cause weakening of the venous wall, resulting in dilation and a tortuous appearance. The extracellular matrix (ECM) is crucial for the structural integrity of the vessel wall. Recent advances in proteomics techniques make it feasible to characterize the composition of the ECM and its remodelling in disease.^4–6^ Our group has previously introduced novel proteomics methods for the analysis of ECM in clinical samples: (i) A proteomics method to characterize the arterial ECM resulting in the discovery of novel glycoproteins in the human aorta^7^ and (ii) a substrate-guided proteomics approach to relate the activity of specific proteases to ECM degradation products in arteries and to identify novel MMP targets.^6,8^ No systematic analysis of pathological ECM remodelling in response to venous hypertension has been performed thus far.

In the present study, we explore the differences in the ECM composition of normal saphenous veins (NSV) and varicose saphenous veins (VSV), and provide unprecedented insights into the expression and degradation of structural and regulatory components of the vascular ECM in varicosis.

## 2. Methods

See http://cardiovascres.oxfordjournals.org for an expanded description of the methods.

### 2.1 Subjects

The investigation conforms with the principles outlined in the Declaration of Helsinki. Following approval from the Institutional Review Board at the Massachusetts General Hospital (IRB; protocol 2010P002776) and written informed consent, varicose great saphenous vein specimens (*n* = 6) were surgically removed during stab incision phlebectomy at the knee level. Normal saphenous vein specimens (*n* = 6) were obtained by surgical resection in patients undergoing coronary artery bypass surgery; the segment of the saphenous vein near the knee level was collected for analysis from each patient. Clinical characteristics for all patients recruited for this study are summarized in Supplementary material online, *Table S1*. Both cohorts were comparable with regards to age and gender. All vein samples were immediately processed following surgical removal, i.e. adipose tissue was trimmed and blood rinsed using cold saline solution. Segments of the vein tissue from each patient were either placed in formalin for subsequent histological analysis or snap frozen in liquid nitrogen and stored at −80°C for future analysis.

### 2.2 ECM protein enrichment

Vein tissues were subjected to our sequential extraction procedure as previously described.^7^ In brief, vessels were washed briefly with cold PBS, incubated in 0.5 M sodium chloride (NaCl) plus proteinase and phosphatase inhibitors for 4 h, followed by decellularization using 0.08% SDS and extraction of ECM proteins using 4 M guanidine hydrochloride (GuHCl) for 48 h before processing for proteomics analysis. Details are given in the Supplementary material online.

### 2.3 Mass spectrometry analysis

Before mass spectrometry analysis, tryptic peptides from processed NaCl and GuHCl extracts were separated on a nanoflow LC system (Thermo Fisher Scientific, Ultimate 3000 RSLC nano). For gel-LC-MS/MS analysis, spectra were collected from an Orbitrap mass analyser (LTQ-Orbitrap XL, Thermo Fisher Scientific) using full ion scan mode over the mass-to-charge (*m/z*) range 450–1600. MS/MS was performed on the top six ions in each MS scan using the data-dependent acquisition mode with dynamic exclusion enabled. For in-solution LC-MS/MS analysis, spectra were collected from a Q Exactive Plus instrument (Thermo Fisher Scientific) using full MS mode over the mass-to-charge (*m/z*) range 350–1600. MS/MS was performed on the top 15 ions in each MS scan using the data-dependent acquisition mode with dynamic exclusion enabled. For multiple reaction monitoring (MRM), the column was coupled to a triple quadrupole mass spectrometer (TSQ Vantage, Thermo Fisher Scientific) for scheduled measurement. Skyline software (version 2.6, MacCoss Lab Software) was used to generate the transition list with predicted collision energies as well as optimized transitions and retention times. A minimum of four transitions were selected per peptide. Further details are given in the Supplementary material online.

### 2.4 Cell culture of human saphenous vein smooth muscle cells

Samples of saphenous veins were collected from patients undergoing elective coronary artery bypass graft surgery (*n* = 6) at Leeds General Infirmary. Saphenous vein smooth muscle cells (SMCs) were extracted and expanded as detailed in the Supplementary material online. SMCs were seeded into 6-well tissue culture plates. Twenty-four hours after seeding, cells were serum starved for 1 h. All treatments were for 24 h, and the following stimuli were diluted in serum-free DMEM at the concentrations indicated: human transforming growth factor beta-1 (TGFβ-1, R&D systems) 10 ng/mL, recombinant mouse TNFα (R&D systems) 100 ng/mL, and a synthetic peptide corresponding to human angiotensin II (Ang II, Sigma-Aldrich) 100 ng/mL. Changes in gene expression after stimulation were measured by RT–qPCR using probes against the corresponding human targets (for a list of all probes used, see Supplementary material online, *Table S2*).

### 2.5 *In vitro* digestion of NSV

NSV (*n* = 5) were digested with recombinant human chymase (1 µg/mL, R&D Systems) or tryptase β1 (250 ng/mL, R&D Systems) according to the protocol supplied by the manufacturer. Controls were incubated in enzyme buffer without enzyme (*n* = 5). After a buffer exchange, the digests were analysed by proteomics as previously described.^8^ The proteins released after digestion were concentrated and separated by SDS–PAGE. Gel separation of proteins facilitates the investigation of protein degradation as protein laddering results in peptide identifications across multiple gel bands. The entire lane was excised, subjected to in-gel tryptic digestion, and analysed by LC-MS/MS. Evidence of degradation (*P* < 0.05) was further validated by western blotting.

### 2.6 Statistical analysis

The adjusted total ion current (TIC) was used for quantification of the in-solution digest. Spectral counts were used for the in-gel digests. Unpaired Student's *t-*tests were used to compare protein abundance and gene expression between NSV and VSV. To compare the effect of different treatments on human venous SMCs, an ANOVA with Dunnett's correction was used. Pieces of NSV from five different donors were incubated with recombinant enzymes. Paired Student's *t-*tests were used to compare the release of ECM proteins from the same NSV (*n* = 5) with and without enzymatic digestion. Quantitative heat maps were generated using MultiExperiment Viewer software (MeV v4.9, TM4). For visualization purposes, all TIC values were transformed to N(0,1) for each protein. The relative difference in normalized spectral counts is represented by a colour gradient. No outliers were removed. A *P*-value of <0.05 was considered significant for all tests used. For graphical representation, averages ± S.D. are shown, unless otherwise specified in the corresponding figure legend.

## 3. Results

### 3.1 Clinical samples

**Figure 1:**
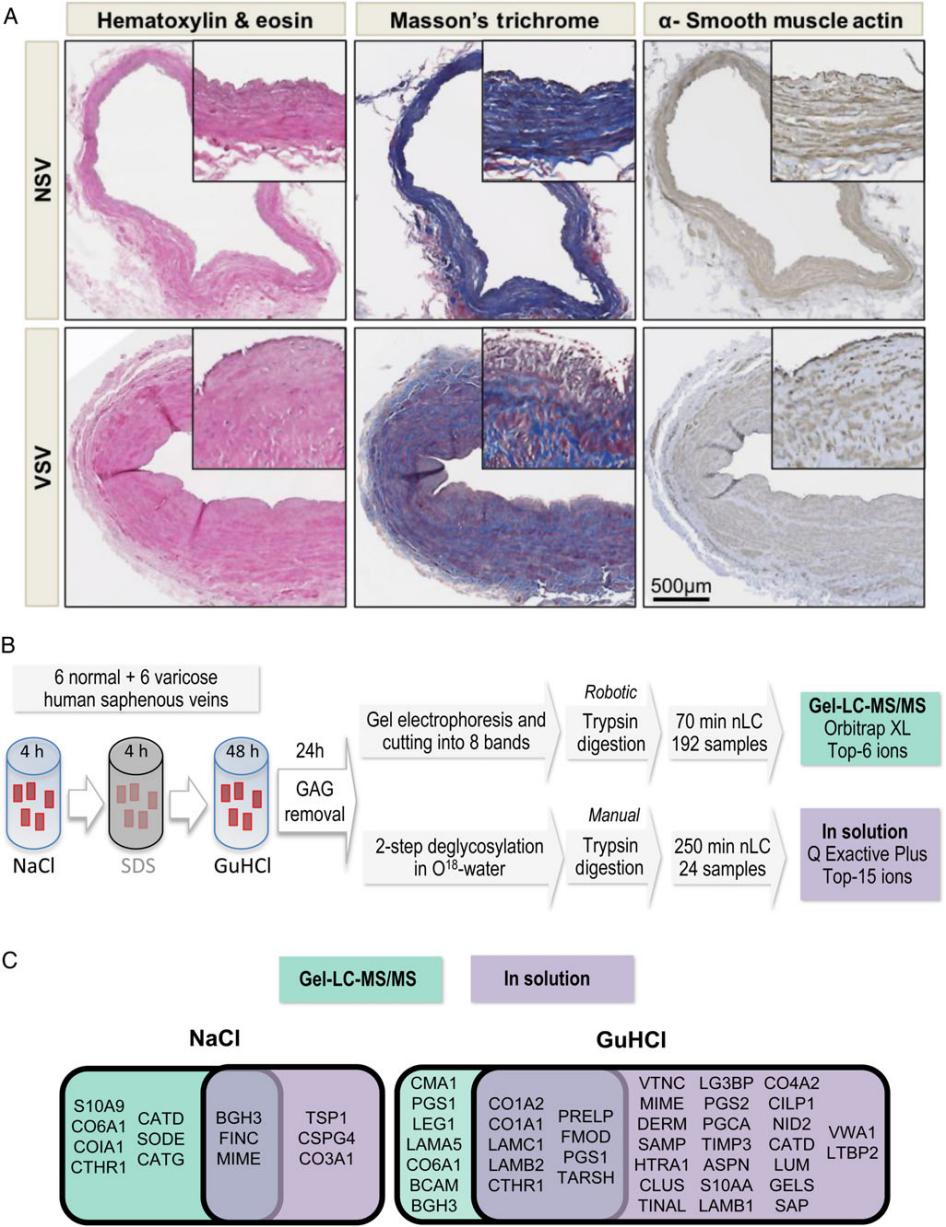
Characterization of human VSV and results from two different proteomics approaches. (*A*) NSV and VSV were stained with haematoxylin and eosin, with Masson's trichrome and with antibodies for α-smooth muscle actin (SMA). (*B*) ECM proteins of NSV (*n* = 6) and VSV (*n* = 6) were obtained using our previously published extraction procedure and analysed by LC-MS/MS after separation by gel-LC-MS/MS or in-solution digestion. (*C*) Comparison of differential protein expression in NSV and VSV by gel- and in-solution LC-MS/MS.

NSV and VSV were used from 12 patients (*n* = 6 per group). On inspection, VSV were abnormal in appearance demonstrating characteristic dilation and tortuosity. NSV were obtained during coronary bypass surgery and showed no tortuosity or luminal dilation on inspection. Electronic medical records of each patient were reviewed for evidence of chronic venous insufficiency including imaging examinations such as ultrasound study of the lower extremities. Patients in the control group did not have a history of chronic venous insufficiency or symptoms of pain, swelling, and/or heaviness in the lower extremities. In contrast, all patients in the VSV group had documented sonographic evidence of chronic venous insufficiency and symptoms of pain, swelling, and heaviness in the lower extremity. The diameter of the VSV collected for analysis at the knee level typically measured 5–6 mm with vein valve closure times ranging from 5 to 7 s indicating significant venous reflux. Both cohorts were comparable with regards to age and gender (see Supplementary material online, *Table S1*). Prior to molecular analysis, tissues obtained from patients were histologically evaluated to confirm the presence of venous disease (*Figure 1A*). VSV demonstrated extensive neointima formation with subendothelial fibrosis, wall thickening, and luminal dilation. In contrast, venous tissue from the NSV group demonstrated normal architecture with no evidence of neointima formation or fibrosis. All tissue sections stained positive for α-smooth muscle cell actin (SMA).

### 3.2 Discovery proteomics of the ECM

Following confirmation of tissue histology, sequential segments of the vein tissue were processed for proteomic analysis (*Figure 1B*). Both VSV and NSV were diced into small pieces and subjected to a three-step extraction procedure as previously described.^7^ Vein samples were consecutively incubated with 0.5 M NaCl, 0.08% SDS, and 4 M GuHCl to decellularize and sequentially extract extracellular space proteins. Subsequently, two proteomics workflows were applied: firstly, NaCl and GuHCl extracts were separated by SDS–PAGE to reduce sample complexity; the entire lane was divided into a series of gel bands; and proteomic analysis was performed on each of them as previously described.^7^ Secondly, NaCl and GuHCl extracts were subjected to an in-solution digest. This MS/MS dataset was deposited in a public repository for mass spectrometry data (http://www.ebi.ac.uk/pride, project accession: PXD002555). The in-solution digest method combined with a faster, more sensitive mass spectrometer resulted in the detection of 152 ECM proteins in the GuHCl extracts and 136 in the NaCl extracts, compared with 90 and 115, respectively, in the gel-LC–MS/MS approach (see Supplementary material online, *Figure S1*, including representative MS spectra).

### 3.3 ECM composition of veins

As expected by our subfractionation procedure, NaCl extracts were enriched with proteins of the extracellular space and newly synthesized ECM proteins, which are not heavily cross-linked in the interstitial matrix (see Supplementary material online, *Table S3*). Fibulins, ECM-associated peptidases, or fibril-associated collagens with an interrupted triple helix (FACITs) were already solubilized by the incubation with 0.5 M NaCl. GuHCl extracts instead contained predominantly less soluble proteins such as network-forming collagens, proteins associated with collagens, laminins, or large aggregating proteoglycans (see Supplementary material online, *Table S4*). Differences in protein identifications between GuHCl and NaCl extracts are highlighted in Supplementary material online, *Figure S2*. Notably, 48 proteins were identified for the first time in the venous ECM (*Table 1*; see Supplementary material online, *Table S5* for an expanded version).

**Table 1:** ECM proteins with differential abundance between NSV and VSV based on in-solution digests

Full name	Uniprot entry	NSV (Av ± SD)	VSV (Av ± SD)	Fold change	*P*-value	Extract
Aggrecan	PGCA_HUMAN	29.9 ± 21.2	1.8 ± 2.9	0.06	0.009	GuHCl
Asporin	ASPN_HUMAN	207.1 ± 132.3	41.0 ± 31.6	0.20	0.014	GuHCl
Biglycan	PGS1_HUMAN	1182.9 ± 205.2	442.9 ± 255.7	0.37	<0.001	GuHCl
Cartilage intermediate layer protein 1	CILP1_HUMAN	5.9 ± 2.0	2.1 ± 2.9	0.35	0.025	GuHCl
Cathepsin D	CATD_HUMAN	1.3 ± 1.3	–	NA	NA	GuHCl
Chondroitin sulfate proteoglycan 4	CSPG4_HUMAN	–	1.2 ± 1.1	NA	NA	NaCl
Clusterin	CLUS_HUMAN	36.4 ± 11.5	11.3 ± 8.9	0.31	0.002	GuHCl
Collagen alpha-1(I)	CO1A1_HUMAN	790.5 ± 277.5	2017.0 ± 585.5	2.55	0.001	GuHCl
Collagen alpha-1(III)	CO3A1_HUMAN	13.7 ± 10.4	3.5 ± 3.1	0.25	0.043	NaCl
Collagen alpha-2(I)	CO1A2_HUMAN	457.9 ± 168.6	1129.7 ± 367.0	2.47	0.002	GuHCl
Collagen alpha-2(IV)	CO4A2_HUMAN	144.7 ± 51.4	84.9 ± 9.1	0.59	0.019	GuHCl
Collagen triple helix repeat-containing protein 1	CTHR1_HUMAN	4.5 ± 4.5	–	NA	NA	GuHCl
Decorin	PGS2_HUMAN	1934.4 ± 590.4	1049.0 ± 259.7	0.54	0.007	GuHCl
Dermatopontin	DERM_HUMAN	425.7 ± 103.5	129.9 ± 54.9	0.31	<0.001	GuHCl
Fibromodulin	FMOD_HUMAN	56.6 ± 32.2	12.6 ± 9.7	0.22	0.009	GuHCl
Fibronectin	FINC_HUMAN	103.1 ± 56.2	31.8 ± 14.9	0.31	0.013	NaCl
Galectin-3-binding protein	LG3BP_HUMAN	2.9 ± 1.3	0.6 ± 0.9	0.19	0.004	GuHCl
Gelsolin	GELS_HUMAN	97.1 ± 58.3	159.7 ± 23.7	1.65	0.035	GuHCl
Laminin subunit beta-1	LAMB1_HUMAN	2.9 ± 0.9	4.9 ± 1.4	1.67	0.018	GuHCl
Laminin subunit beta-2	LAMB2_HUMAN	105.9 ± 40.9	171.5 ± 45.6	1.62	0.026	GuHCl
Laminin subunit gamma-1	LAMC1_HUMAN	80.9 ± 34.9	156.4 ± 31.5	1.93	0.003	GuHCl
Latent-TGFβ-binding protein 2	LTBP2_HUMAN	4.8 ± 2.0	1.5 ± 1.1	0.32	0.007	GuHCl
Lumican	LUM_HUMAN	1577.2 ± 436.8	979.7 ± 411.1	0.62	0.035	GuHCl
Metalloproteinase inhibitor 3	TIMP3_HUMAN	3.3 ± 2.4	0.1 ± 0.4	0.05	0.010	GuHCl
Mimecan	MIME_HUMAN	633.1 ± 89.0	293.2 ± 78.2	0.46	<0.001	GuHCl
Mimecan	MIME_HUMAN	12.1 ± 7.9	4.6 ± 1.7	0.38	0.047	NaCl
Nidogen-2	NID2_HUMAN	29.7 ± 14.7	14.3 ± 3.2	0.48	0.031	GuHCl
Prolargin	PRELP_HUMAN	1203.6 ± 293.0	484.1 ± 261.4	0.40	0.001	GuHCl
Prosaposin	SAP_HUMAN	3.5 ± 1.1	2.0 ± 1.0	0.58	0.040	GuHCl
Protein S100-A10	S10AA_HUMAN	18.3 ± 5.9	8.2 ± 6.2	0.45	0.017	GuHCl
Serine protease HTRA1	HTRA1_HUMAN	29.6 ± 13.6	4.0 ± 5.3	0.14	0.002	GuHCl
Serum amyloid P-component	SAMP_HUMAN	107.2 ± 41.8	10.7 ± 12.5	0.10	<0.001	GuHCl
Target of Nesh-SH3	TARSH_HUMAN	155.6 ± 86.9	72.7 ± 27.1	0.47	0.050	GuHCl
TGFß-induced protein ig-h3	BGH3_HUMAN	15.2 ± 4.0	79.6 ± 39.4	5.25	0.003	NaCl
Thrombospondin-1	TSP1_HUMAN	16.7 ± 10.4	36.1 ± 8.1	2.17	0.005	NaCl
Tubulointerstitial nephritis antigen-like	TINAL_HUMAN	122.0 ± 35.5	58.8 ± 21.6	0.48	0.004	GuHCl
Vitronectin	VTNC_HUMAN	23.3 ± 5.5	4.4 ± 3.6	0.19	<0.001	GuHCl
von Willebrand factor A domain-containing protein 1	VWA1_HUMAN	13.9 ± 9.6	4.6 ± 2.9	0.33	0.048	GuHCl

Average ± standard deviation (Av ± SD) based on total ion current (TIC × 10 to the power of 6).

*P*-values were derived from unpaired Student's *t*-tests with unequal variance (Note that every time protein expression in the majority of samples from 1 of the 2 groups compared was undetectable, the *t*-test was not performed; hyphens (-) denote these proteins without detection in one of the groups). NA denotes ‘not applicable’.

### 3.4 Proteomics comparison of NSV and VSV

**Figure 2:**
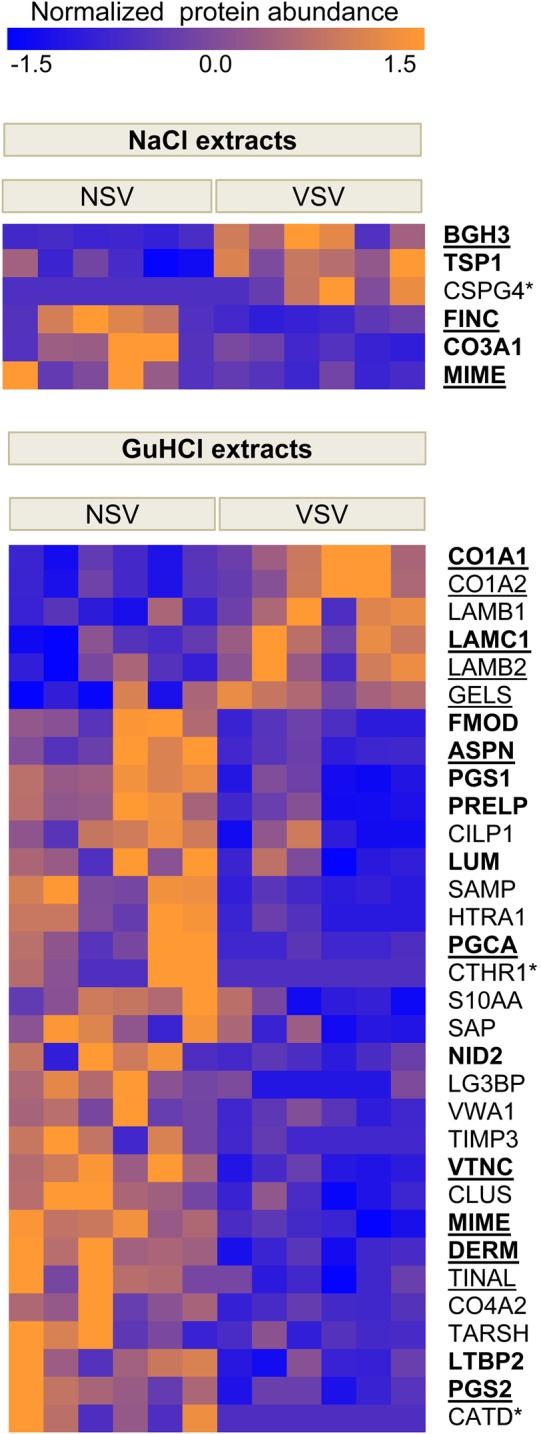
Proteomics revealed a distinct ECM composition in NSV and VSV. Heat maps for NaCl and GuHCl extracts in NSV and VSV based on normalized protein abundance to allow for representation of proteins with different abundances (*n* = 6 per group). Underlined proteins were validated by either MRM or western blot analysis. Proteins in bold were analysed at the transcriptional level by RT–qPCR. Proteins without detection for one of the groups are labelled with an asterisk (*). For detailed information, refer to *Table 1*. Statistical significance (*P* < 0.05) was calculated using Student's *t*-tests.

Overall, more differentially expressed proteins were identified in the in-solution digest (*Table 1*; see Supplementary material online, *Table S5* for an expanded version) than in the gel-LC-MS/MS approach (see Supplementary material online, *Table S6*), and more proteins were differentially expressed in the GuHCl extracts than in the NaCl extracts (*Figure 1C*). In the NaCl extracts, TGFβ-induced protein ig-h3 (BGH3), a marker of TGFβ activity, and thrombospondin-1 (TSP1), a potent activator of TGFβ, were up-regulated in VSV. In the GuHCl extracts, collagen I chains α-1 and α-2 (CO1A1 and CO1A2) and laminins β-1, β-2, and γ-1 (LAMB1, LAMB2, and LAMC1) were increased. Most regulatory ECM components, however, were reduced, in particular small leucine-rich proteoglycans (SLRPs) that are responsible for collagen fibril formation, and important for the proper formation of the vascular ECM (*Figure 2*).

**Figure 3:**
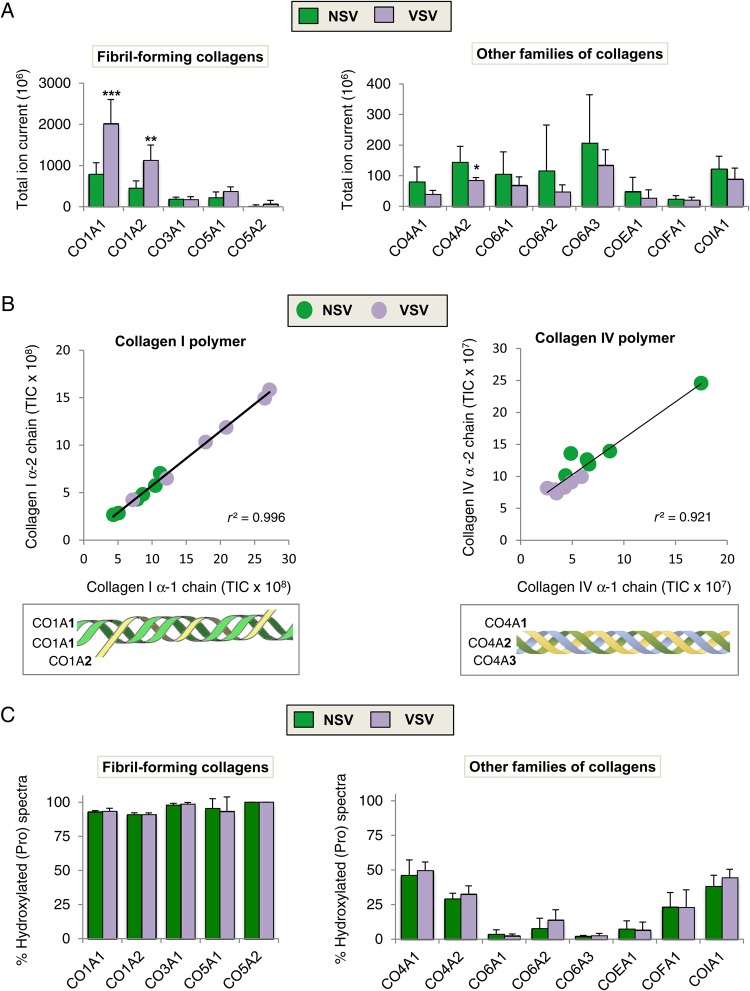
Alterations in collagen composition in VSV. (*A*) Abundance of fibril-forming collagens compared with other families of collagens detected in GuHCl extracts from NSV (*n* = 6) and VSV (*n* = 6). For visualization purposes, the total ion current (TIC) for CO5A1 and CO5A2 was scaled up 10-fold in the left panel. CO6A1 and CO6A3 were scaled down five-fold in the right panel. (*B*) Different chains form cross-linked collagen polymers within vascular tissues. Scatter plots for protein abundance (expressed as TIC) demonstrate high correlation for different chains of the same collagen. (*C*) Proportion of hydroxylated prolines in collagens from NSV compared with VSV. A much higher proportion is observed for fibril-forming collagens compared with other families. Dysregulation in collagen content in VSV did not affect the rate of collagen hydroxylation. Statistical significance was calculated using Student's *t*-tests. * denotes *P* < 0.05, **P < 0.01, ***P < 0.001.

All collagens form triple helices, but resistance to tensile stress is of particular importance in collagens that contribute to fibre formation. Among these fibrillar collagens, collagens I, III, and V are abundant in the vasculature.^7^ The increase of CO1A1 and CO1A2 (i.e. fibril-forming collagens) in VSV was in contrast to other families of collagen, which were generally decreased (*Figure 3A*). Evidence for the quantitative accuracy of our proteomic method is the strong correlation (*r* > 0.9) between the abundance of different collagen subunits (*Figure 3B*).

Hydroxylation of collagen on prolines within X-Pro-Gly triplets is a key process during fibre formation.^9^ On average, 96% of peptides belonging to fibrillar collagens contained this post-translational modification. Hydroxylation was less abundant in other collagens (*Figure 3C*). Although detectable, hydroxylated Pro-Hyp-Gly sequences, which are targets for prolyl-3-hydroxylase,^9^ and hydroxylation of lysine^10^ were less common (see Supplementary material online, *Figure S3*). No differences were observed in collagen hydroxylation between NSV and VSV.

### 3.5 Validation with targeted proteomics

**Figure 4:**
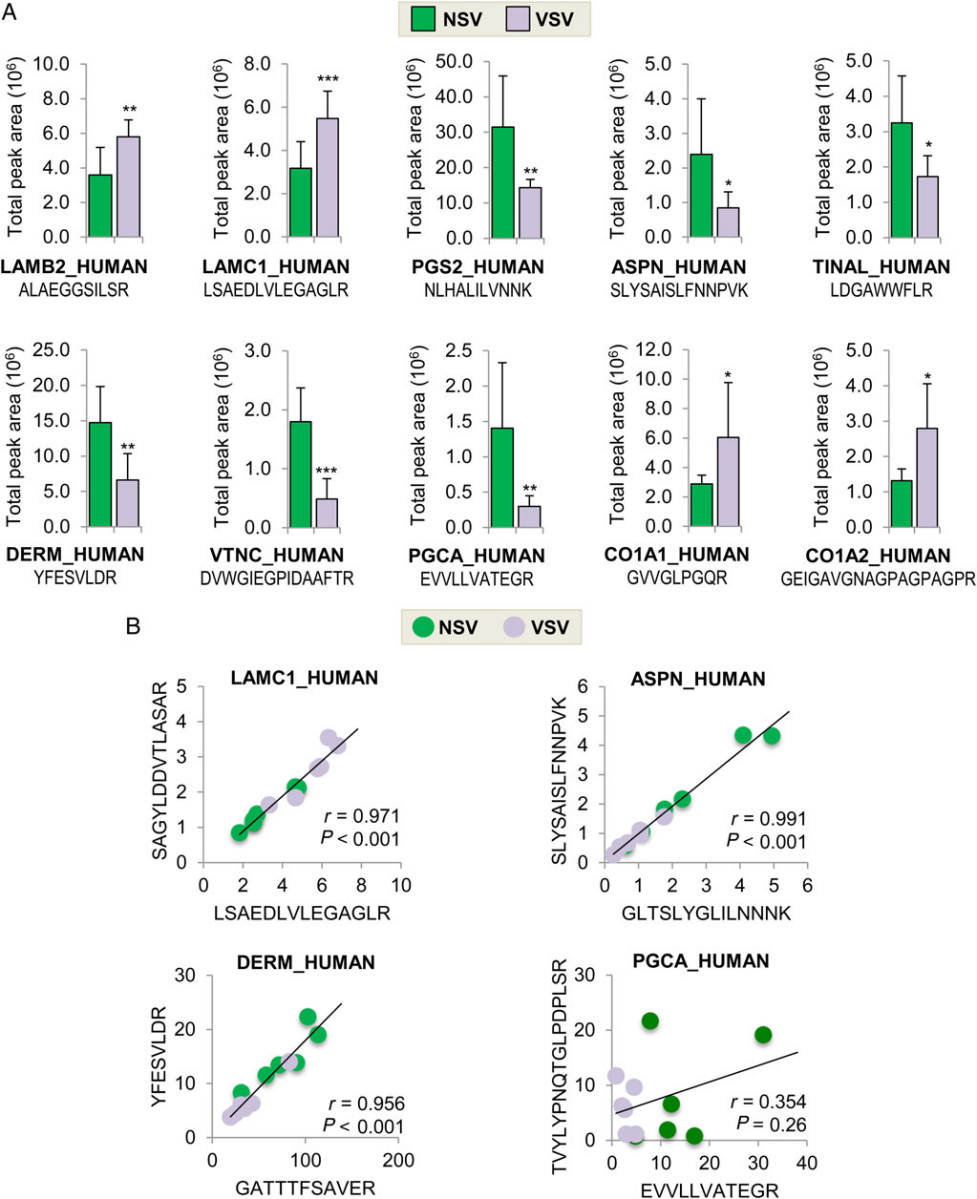
Targeted proteomics (MRM analysis) to validate the proteomics findings. (*A*) MRM assays were used to validate differential expression of additional ECM proteins in NSV (*n* = 6) and VSV (*n* = 6). LAMB2, laminin subunit β2; LAMC1, laminin subunit γ1; PGS2, decorin; ASPN, asporin; TINAL, tubulointerstitial nephritis antigen-like protein; DERM, dermatopontin; VTNC, vitronectin; PGCA, aggrecan; CO1A1 and CO1A2, collagen α1 (I) and α2(I) chains. (*B*) MRM analysis for two peptides of the same protein demonstrated high correlation, which is an indication of robustness of our measurements. Only aggrecan (PGCA) showed poor correlation. Statistical significance was calculated using Student's *t*-tests. * denotes *P* < 0.05, **P < 0.01, ***P < 0.001.

We took advantage of our MS data from different cardiovascular territories^5–8^ and selected additional proteotypic peptides for targeted MRM analysis (see Supplementary material online, *Figure S4* and *Table S7*) to confirm the increase in LAMB2 and LAMC1, and the loss of decorin (PGS2), asporin (ASPN), tubulointerstitial nephritis antigen-like protein (TINAL), dermatopontin (DERM), vitronectin (VTNC), and aggrecan (PGCA) in VSV. The aforementioned increase of CO1A1 and CO1A2 in VSV was also confirmed by MRM analysis (*Figure 4A*). The accuracy of our MRM method was demonstrated by selecting two different proteotypic peptides for the same protein (*Figure 4B)*. For most proteins, the correlation between the two different peptides for the same protein was high (i.e. *r* > 0.95). An exception was aggrecan, probably because the selected peptides were on opposite sites of a common aggrecanase cleavage site.

### 3.6 Validation by independent techniques

**Figure 5:**
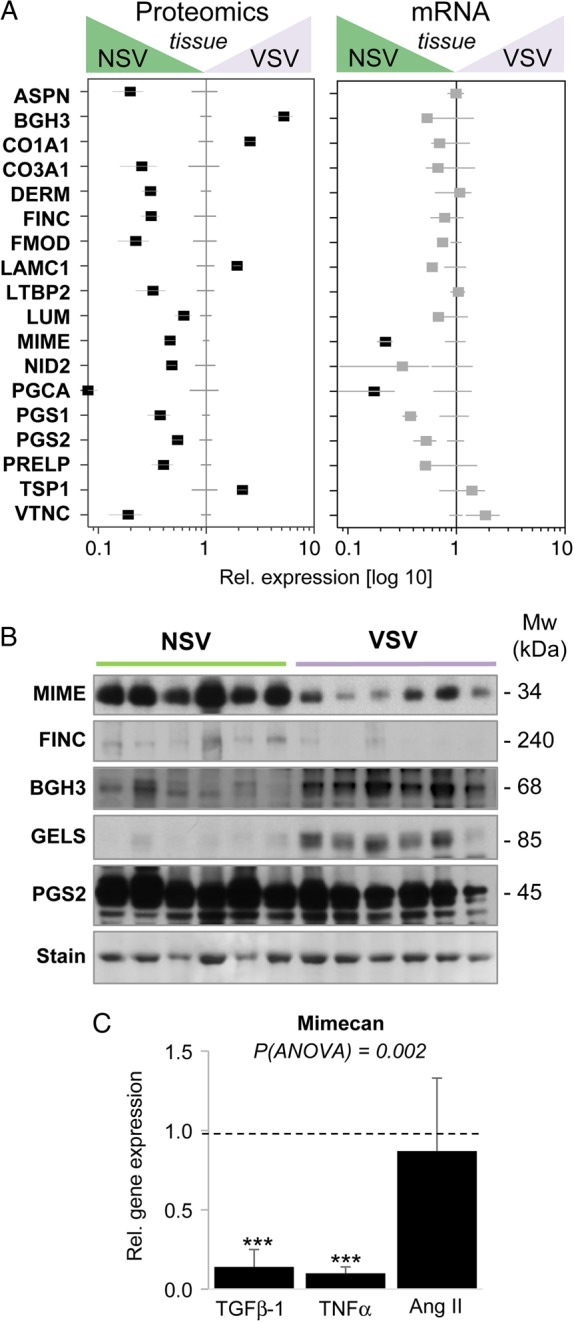
Discrepancies between protein abundance in the ECM and corresponding gene expression levels. (*A*) Relative gene expression of ECM targets (*Figure 2*) in NSV and VSV. Protein abundance levels detected after proteomics analysis of NSV and VSV are shown for comparison (left plot). To compare transcripts and proteins with disparate basal values, protein abundance and gene expression levels were set to ‘1’ for NSV. Horizontal lines represent the SEM for NSV and VSV (*n* = 6 per group). Black squares represent significant (*P* < 0.05) changes as determined by Student's *t*-test. (*B*) Immunoblotting for mimecan (MIME), fibronectin (FINC), TGFβ-induced protein ig-h3 (BGH3), gelsolin (GELS), and decorin (PGS2). (*C*) Venous human SMCs from six donor patients incubated with TGFβ-1 and TNFα showed reduced mimecan expression. Incubation with Ang II did not affect mimecan expression. Levels of expression for unstimulated control cells are represented by the dashed line. Statistical significance was calculated using an ANOVA test with Dunnett's correction. *** denotes *P* < 0.001.

Gene expression analysis in the same tissues demonstrated that most changes in ECM protein abundance were not accompanied by corresponding changes in gene expression (*Figure 5A*). Only the down-regulation of mimecan (MIME) and aggrecan (PGCA) in VSV was observed at the protein as well as the transcript level. The loss of fibronectin (FINC, *Table 1*) and galectin-1 in VSV (see Supplementary material online, *Table S6*) was corroborated by immunohistochemistry (see Supplementary material online, *Figure S5*). Additional immunoblots were performed for mimecan, fibronectin, TGFβ-induced protein ig-h3, gelsolin (GELS), and decorin to confirm the changes in protein abundance (*Figure 5B*; see Supplementary material online, *Figure S5* for quantification by densitometry). The down-regulation of mimecan was further validated by using venous human SMCs stimulated with TGFβ-1, TNFα, or Ang II. TGFβ-1 and TNFα, but not Ang II, were able to induce a repression of mimecan gene expression (*Figure 5C*).

### 3.7 Proteolytic degradation of the venous ECM

**Figure 6:**
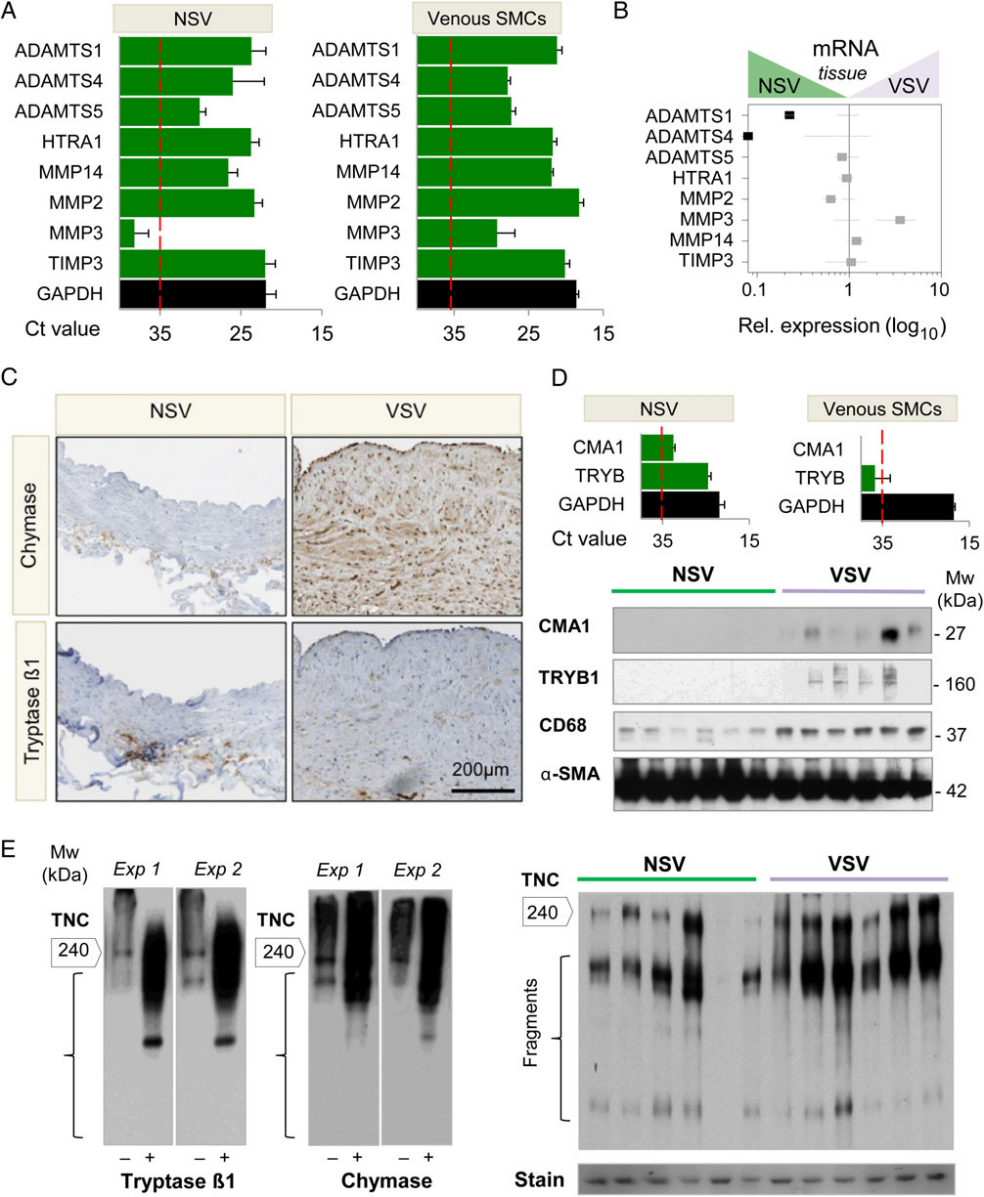
Protease activity as contributor to ECM remodelling in VSV. (*A*) Ct values for different vascular proteases in NSV (*n* = 6) and venous SMCs (*n* = 6) as determined by RT–qPCR. A Ct value >35 was considered undetectable. ADAMTS, a disintegrin metalloproteinase with thrombospondin domains; HTRA1, serine protease HTRA1; MMP, matrix metalloproteinase, TIMP, tissue inhibitor of matrix metalloproteinase; GAPDH, glyceraldehyde-3-phosphate dehydrogenase. (*B*) A down-regulation for the aggrecanases ADAMTS1 and ADAMTS4 was observed in VSV. Data points are averages ± SEM. Black squares represent significant (*P* < 0.05) changes as determined by Student's *t*-test. (*C*) Distribution of chymase and tryptase β1 in NSV and VSV. Note the extensive cellular staining for chymase. A patchy, less prominent distribution was observed for tryptase β1. (*D*) Chymase (CMA1) and tryptase β1 (TRYB) were expressed in vein tissues but were undetectable in cultured venous SMCs. Western blot analysis of NSV and VSV confirmed higher abundance of chymase and tryptase β1 in VSV. This paralleled an increased detection for CD68 and decrease of α-SMA. (*E*) NSV samples (*n* = 5) were split into two halves and incubated with the two proteases or the corresponding buffers only, before western blot analysis. An example is shown for two experiments. Incubation with the proteases led to detection of a degradation pattern in tenascin C (TNC). Boxed numbers indicate the expected size (kDa) for the full-length protein. Protein degradation products for tenascin were observed in VSV similar to the observations in the adjacent panel. Ponceau S staining was used as a loading control.

Apart from collagen I and laminins, many ECM proteins were down-regulated in the GuHCl fraction of VSV compared with NSV, including the large aggregating proteoglycan aggrecan. Gene expression analysis demonstrated that aggrecanases from the ADAMTS (a disintegrin metalloproteinase with thrombospondin domains) family (ADAMTS1, 4, 5) are expressed in veins as well as venous SMCs (*Figure 6A*). Along with aggrecan, transcript levels for ADAMTS1 and ADAMTS4 were reduced in VSV (*Figure 6B*). Other proteases screened and the protease inhibitor TIMP3 did not show a significant change.

Thus, we searched for additional proteases in our proteomics dataset. Two mast cell proteases were identified in VSV: chymase and tryptase β1. Both tryptase β1 and chymase could be localized in the neointima of VSV. However, the staining pattern revealed a different localization within the vessel wall. Tryptase β1-positive cells were mainly localized in the adventitia of the vessel wall, whereas chymase staining was seen throughout the vessel wall (*Figure 6C* and see Supplementary material online, *Figure S6*). Gene expression analysis showed that both chymase and tryptase β1 were expressed in tissue samples but not in cultured human venous SMCs (*Figure 6D,* top panel), even when stimulated with TGFβ-1, TNFα, and Ang II (data not shown). Instead, the increased abundance of these mast cell proteases in VSV was paralleled by an increase in CD68, a marker for the various cells of the macrophage lineage, and a decrease of α-SMA (*Figure 6D*, lower panel; see Supplementary material online, *Figure S6* for quantification by densitometry).

To investigate the effect in venous tissue of tryptase β1 and chymase, NSV macroscopically free of vascular disease were subjected to an overnight digestion with tryptase β1 and chymase. NSV incubated in enzyme buffer-only were used as controls. The proteins released after digestion were analysed by gel-LC-MS/MS (see Supplementary material online, *Figure S7*). Extracellular proteins detected after chymase and tryptase β1 digestion of NSV are listed in Supplementary material online, *Table S8* and *Table S9*, respectively. The effect of the treatment with tryptase β1 and chymase on the distribution of various protease targets is presented in the Supplementary material online, *Figure S8*. The in-gel approach allowed assignments of fragments from ECM proteins identified in bands lower than their expected molecular weights (Mw) resulting in higher spectral counts or a shift in their Mw distribution. Only for small proteins like galectin-1 (14 kDa), fragments were not detected upon digestion, probably due to migration ahead of the gel front. Notably, the effect of tryptase β1 on the venous ECM was generally more pronounced (see Supplementary material online, *Table S10*). Basement membrane (BM)-associated proteins, such as collagen VI, perlecan, and tenascins, however, were predominantly affected by digestion with chymase. In contrast, no effect was observed on CO1A1 and CO1A2, suggesting that increased chymase levels cannot attenuate collagen I accumulation in VSV. For independent validation, we performed immunoblotting for tenascin C on the conditioned medium after digestion with tryptase β1 and chymase. Degradation was observed following incubation with both proteases (*Figure 6E*, left panel), and a similar degradation pattern was observed in VSV (*Figure 6E,* right panel). Thus, besides changes in gene expression, remodelling in VSV involves proteolysis of ECM components.

## 4. Discussion

**Figure 7:**
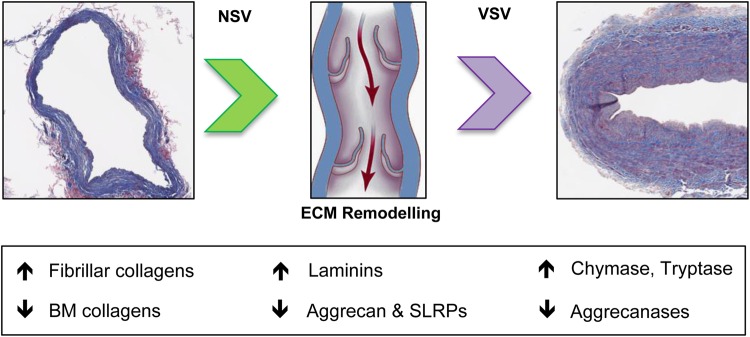
Summary of the proteomics findings. ECM remodelling is a hallmark of VSV formation. Differences detected in the present study reflect a combination of changes in protein abundance, gene expression, and proteolytic activity.

In continuation of our previous work on applying proteomics to study the cardiovascular ECM,^5–8^ this is the first proteomics analysis of the ECM in human veins that describes the remodelling process of the venous ECM, a critical step in the development of varicosis. The key proteomic findings are summarized in *Figure 7*.

### 4.1 ECM remodelling in veins

Venous hypertension in the hepatic, splanchnic, and peripheral circulation affects a large population of patients presenting with liver, renal, and cardiovascular disease. Although the causes of venous hypertension may be multifactorial, epigenetic factors at the level of the venous wall may also be involved in mediating susceptibility. Varicose veins are among the most prevalent medical problems today with worldwide prevalence estimates of up to 73% in females and 56% in males.^11^ It is associated with significant morbidity and has a considerable economic impact causing an estimated loss of 2 million workdays and costing $3 billion to treat in the USA alone.^12^ The pathogenesis of varicose veins is associated with many predisposing factors including pregnancy, trauma, obesity, and prolonged standing. However, it is becoming increasingly apparent that its development is more complex, likely involving inborn, epigenetic, or local genetic traits that probably play key roles in increasing susceptibility to developing varicose veins.^3^

Until now, a comprehensive analysis of ECM components comprising NSV and VSV has not been performed. In recent years, our group has used proteomics to study ECM remodelling in different cardiovascular diseases, including in aortic specimen.^5,6,13^ Compared with other tissues, the decellularization step is a key to reduce complexity and minimize carryover from cell- and blood-derived proteins in cardiovascular tissues.^14^ A caveat of the gel-LC-MS/MS approach is the fact that very large ECM proteins may not migrate into the gel.^15^ On the other hand, the presence of these high Mw proteins can mask the presence of less abundant ECM proteins since they generate far more tryptic peptides than low Mw proteins. Similarly, in the present study, both approaches revealed interesting and complimentary protein changes that were validated by independent techniques, including western blot and MRM analysis.

In VSV, the perivascular space is surrounded by ECM proteins. This perivascular cuff is an initial response to an increased mechanical load and is accompanied by collagen deposition. However, the collagen fibres show abnormal distribution and morphology. Immunohistochemical analyses demonstrated that VSV had irregular collagen fibrillogenesis and redundant lamellae of BM material.^16^ Our proteomics results corroborate these histological findings by demonstrating an increase in collagen I chains and laminins in VSV. Other BM proteins and collagen-binding proteins required for assembly, size, and structure of collagen fibres showed a marked reduction in chronic venous insufficiency. TGFβ-induced protein ig-h3, a downstream marker for TGFβ activity, and thrombospondin-1, a key activator of TGFβ, were increased confirming previous reports of exacerbated levels of local TGFβ in VSV.^17^ In addition, our proteomics study revealed a decrease in SLRPs.^18^ All detected SLRPs in venous vessels (decorin, mimecan, biglycan, fibromodulin, prolargin, lumican, asporin, and podocan) have horseshoe shapes and embrace collagen fibres to ensure appropriate calibre, shape, and disposition.^19^ Furthermore, decorin, biglycan, fibromodulin, and asporin bind TGFβ, repressing the activation of pro-fibrotic pathways.^18^ Reduced levels of SLRPs with TGFβ-binding activity and of TGFβ-sequestering proteins such as cartilage intermediate layer protein 1 (CILP1, *Figure 2*) support the notion of exacerbated TGFβ activity in chronic venous insufficiency.^17^ Moreover, our data show that in venous SMCs, expression of other SLRPs such as mimecan is responsive not only to the pro-fibrotic cytokine TGFβ-1, but also to the pro-inflammatory cytokine TNFα, which is known to be elevated in patients with venous ulcers.^20^

### 4.2 Vascular proteases

MMPs with their broad substrate specificity^8^ have been identified as key drivers of ECM degradation, in particular during the earlier stages of venous remodelling.^21^ Decreased protein levels of TIMP3 (i.e. a broad spectrum matrix-associated metalloproteinase inhibitor) detected in our proteomics analysis of the GuHCl extracts (*Table 1*), support the concept of increased proteolytic activity in venous hypertension. TIMP3, however, was not altered at the transcript level (*Figure 6B*) but has previously been implicated in preserving the vascular ECM in arterial hypertension.^22^ Additional metalloproteinases include aggrecanases from the ADAMTS family. The ADAMTS enzymes are secreted, multi-domain zinc metalloendopeptidases with diverse roles in physiopathological remodelling, inflammation, and vascular biology.^23^ Interestingly, aggrecan, a major ECM component of cartilage, has previously been identified in aortic tissue.^7^ Negatively charged glycans on the surface of aggrecan attract water and therefore confer resistance to compression. To our knowledge, the present study is the first to report the identification of aggrecan in venous tissue. Expression of aggrecan in VSV was reduced at the protein and transcript levels in comparison to NSV. The loss of aggrecan was accompanied by a reduction of aggrecanase expression (ADAMTS1 and ADAMTS4).

Our proteomics analyses returned additional proteases, including chymase and tryptase β1. These serine proteases are commonly attributed to secretory granules of mast cells and promote inflammation and ECM remodelling.^24,25^ Although one study reported no significant difference in the mean mast cell density in the wall of varicose and non-dilated veins,^26^ several groups have demonstrated that mast cells are a predominant cell type in VSV.^27^ Intriguingly, chymase staining in VSV rarely co-localised with tryptase β1 (*Figure 6C*). The presence of a vascular SMC-expressed chymase has previously been reported in arteries of spontaneously hypertensive rats.^28,29^ While the observed staining pattern was suggestive of an ubiquitous presence of chymase in VSV, including SMCs, the lack of chymase expression in cultured venous human SMCs is evidence to the contrary, and stimulation with TGFβ-1, Ang II, and TNFα failed to induce expression (data not shown). Although we observed increased abundance of the macrophage lineage marker CD68 in VSV, a recent paper by Shankman *et al.*^30^ demonstrated that expression of CD68 and other non-SMC markers can be induced in SMCs within atherosclerotic plaques. Thus, lineage tracing rather than co-staining is required to establish cellular origin in diseased tissues.

Apart from a chymase-dependent Ang II-generating pathway in the vessel wall,^28,31^ there are several other mechanisms by which chymases may impact on blood vessels and in particular affect SMCs.^32^ For example, Leskinen *et al.*^33,34^ demonstrated that chymase is pro-apoptotic for vascular SMCs by degrading the pericellular ECM component fibronectin, causing disruption of focal adhesion complexes and Akt dephosphorylation, which are necessary for cell adhesion and survival. Similarly, we have observed fibronectin degradation and preferential release of BM proteins by chymase digestion of NSV. On the other hand, there are endogenous protease inhibitors in the interstitial fluid that would inhibit chymase activity.^35^ The importance of proteolytic activity for the wall stress-induced maladaptive venous remodelling has been highlighted by a recent study; inhibition of the 26S proteasome using bortezomib led to a stabilization of the quiescent SMC phenotype. Bortezomib inhibits the chymotrypsin-like activity of the 26S proteasome, and transdermal administration diminished venous SMC proliferation by 80% and inhibited varicose-like venous remodelling in a mouse model.^36^ Notably, chymases have chymotrypsin-like substrate specificity.

### 4.3 Strengths and limitations

A strength of the study is the use of well-characterized patient samples. A caveat of working with patient specimens is that only end-stage disease can be assessed. Therefore, future studies to clarify the temporal expression and activity patterns of vascular proteases will be required. To that end, animal models offer unique insights into disease progression. Although mast cells have been shown to play detrimental roles in the pathogenesis of both atherosclerosis^37^ and abdominal aortic aneurysm,^38^ mast cell chymase can also prevent an exaggerated SMC expansion.^25,34^ Further studies are required to explore the role and cellular localization of chymase in VSV. Moreover, our proteomics workflow targets extracellular proteins, but released proteins do not necessarily reflect intracellular protein concentrations. For example, increased levels of cathepsins have been observed in VSV.^39^

In conclusion, varicosis is intimately related to dynamic changes in the vascular ECM and its associated proteins. The present proteomics study provides the first comprehensive analysis of the ECM remodelling processes in response to venous hypertension.

## Supplementary material

Supplementary material is available at *Cardiovascular Research* online.

## Funding

This work was supported by the American College of Phlebology (R.O.) and the National Institute for Health Research (NIHR) Biomedical Research Centre based at Guy's and St Thomas’ NHS Foundation Trust and King's College London in partnership with King's College Hospital and an excellence initiative (Competence Centers for Excellent Technologies - COMET) of the Austrian Research Promotion Agency FFG: ‘Research Center of Excellence in Vascular Ageing – Tyrol, VASCage’ (K-Project Nr. 843536) funded by the BMVIT, BMWFW, the Wirtschaftsagentur Wien, and the Standortagentur Tirol. H.A. is funded by Massachusetts General Hospital, Department of Surgery, Division of Vascular and Endovascular Surgery, Henry & Nod Meyer Research Fund. M.M. is a Senior Fellow of the British Heart Foundation.
